# Association Between Lifestyle at Different Life Periods and Brain Integrity in Older Adults

**DOI:** 10.1212/WNL.0000000000213347

**Published:** 2025-02-07

**Authors:** Anne-Laure Turpin, Francesca Felisatti, Léa Chauveau, Sacha Haudry, Florence Mézenge, Brigitte Landeau, Denis Vivien, Vincent De La Sayette, Gaël Chételat, Julie Gonneaud, for the Medit-Ageing Group

**Affiliations:** 1 Normandy University, UNICAEN, INSERM, U1237, PhIND “Physiopathology and Imaging of Neurological Disorders”, NeuroPresage Team, GIP Cyceron, Caen, France;; 2 Normandy University, UNICAEN, INSERM, U1237, PhIND “Physiopathology and Imaging of Neurological Disorders”, Institut Blood & Brain @ Caen, GIP Cyceron, France;; 3 Département de Recherche Clinique, CHU Caen-Normandie, France;; 4 Normandie University, UNICAEN, PSL Université, EPHE, INSERM, U1077, CHU de Caen, GIP Cyceron, NIMH, Pôle des Formations et de Recherche en Santé, Caen, France; and; 5 Service de Neurologie, CHU de Caen, France.

## Abstract

**Background and Objectives:**

Lifestyle behaviors, including engagement in complex mental activities, have been associated with dementia risk and neuroimaging markers of aging and Alzheimer disease. However, the life period(s) at which lifestyle factors have the greatest influence on brain health remains unclear. Our objective was to determine the relative influence of lifestyle (i.e., engagement in complex mental activities) at different life periods on older adults' brain health.

**Methods:**

This observational study included community-dwelling cognitively unimpaired seniors (older than 65 years) from the Age-Well randomized controlled trial (Caen, France). All participants completed at baseline the Lifetime of Experiences Questionnaire, assessing engagement in complex mental activities during young adulthood (13–30 years: LEQ-young), midlife (30–65 years: LEQ-midlife), and late-life (older than 65 years: LEQ-late). LEQ scores were divided into specific and non-specific activities. Multiple regressions were conducted including LEQ scores at the 3 life periods (same model) to predict gray matter volume (GMv; structural-MRI), glucose metabolism (fluorodeoxyglucose-PET), perfusion (early-Florbetapir-PET), or amyloid burden (late-Florbetapir-PET), both in AD-signature regions and voxel-wise (significance for voxel-wise analyses: *p* < 0.005_uncorrected_, k > 100). Correlations between LEQ and neuroimaging outcomes were then compared between (1) life periods and (2) specific and non-specific activities. Analyses were controlled for age and sex.

**Results:**

In 135 older adults (mean age = 69.3 years; women = 61.5%), no associations were found within AD-signature regions (all *p* > 0.25). Voxel-wise analyses revealed no association between LEQ-young and neuroimaging. LEQ-midlife showed stronger voxel-wise associations than the other periods with GMv, notably in the anterior cingulate cortex, and with amyloid burden in the precuneus. These correlations were stronger for the LEQ-midlife specific (i.e., occupation) than the non-specific subscore (GMv: z = 3.25, *p* < 0.001, 95% CI [0.1292–0.5135]; amyloid: z = −1.88, *p* < 0.05, 95% CI [−0.3810 to −0.0113]). LEQ-late showed stronger voxel-wise associations than the other periods with perfusion and glucose metabolism in medial frontal regions. The correlation of perfusion with LEQ-late was stronger for non-specific than specific subscore (z = 2.88, *p* < 0.01, 95% CI [0.0894–0.4606]).

**Discussion:**

Lifestyle at different life periods may have complementary benefits on brain health in regions related to reserve/resilience in aging. While past (midlife) engagement could promote resistance against structural/pathologic alterations, current (late-life) engagement could enhance cognitive reserve. Future larger longitudinal studies should explore mechanisms by which lifestyle promotes reserve.

## Introduction

Aging is a complex physiologic process occurring throughout life, affecting the brain and increasing the risk of dementia, including Alzheimer disease (AD). However, there is a large heterogeneity in brain aging, partly because of differences in resilience mechanisms.^1,2^ These include differences in (1) cognitive reserve (CR), the brain's ability to cope with cerebral damage using more efficient or flexible cognitive processes and brain networks, (2) brain reserve (BR), reflecting the neurobiological capital allowing to better cope with age-related changes and pathology before symptom onset, or (3) brain maintenance (BM), which relates to the absence of biological brain changes. These mechanisms may be influenced throughout life by a variety of genetic and environmental factors, including lifestyle. In that context, lifestyle seems as a promising target to promote healthy aging.^3,4^ Consistently, a healthier lifestyle has been associated with greater brain and cognitive outcomes in cognitively unimpaired older adults.^5-7^ The latest Lancet Commission report,^8^ suggesting that about 45% of dementia cases could be prevented by acting on various modifiable risk factors, proposes a certain temporality of action for these different factors (e.g., early-life education, midlife cardiovascular risk, late-life social isolation).

This raises the question of the period(s) during which lifestyle could have its greatest benefit on brain health, which would be critical to tailor effective lifestyle-based prevention strategies. Previous studies found associations between lifestyle factors at different life periods and brain outcomes.^9-12^ However, such studies have considered each life period independently from the others and, thus, did not allow to directly compare the effect of life periods between them. Importantly, because lifestyle is likely to correlate throughout the lifespan, not considering the shared variance of lifestyle between life periods prevents drawing any conclusion regarding the relative/specific effect of lifestyle engagement for each life period. In addition, only solitary imaging modalities has been usually investigated in these studies.^13-15^ This gives a fragmented view and limits the ability to address the resilience mechanisms behind theses associations between lifestyle throughout the lifespan and brain integrity. For instance, differences in brain structure and amyloid/tau levels could inform on BR and BM, while differences in brain metabolism/perfusion could inform on CR mechanisms.^16^

This study aims at investigating comprehensively the associations between lifestyle at different life periods and brain markers of aging and AD in cognitively unimpaired older individuals. More specifically, we aim at identifying the relative association of lifestyle engagement in young, mid, and late adulthood with multimodal neuroimaging in older adults, within AD-signature regions and throughout the whole brain by comparing directly the effects of life periods between them. We will also investigate the influence of different types of lifestyle activities on these associations, focusing on the distinction between activities that are specific to a given life period (e.g., education, occupation) vs non-specific ones (e.g., leisure activities). We hypothesized that lifestyle behaviors do not have the same influence on older adults' brain health across the life periods, resulting in different associations between lifestyle and different markers of brain integrity for each period considered. Multimodal neuroimaging should enable us to approach the underlying resilience mechanisms.

## Methods

### Participants

Participants were all included in the Age-Well randomized controlled trial (RCT).^17^ The Age-Well trial is a monocentric RCT that was part of the Medit-Ageing European Project, which aimed at investigating the impact of meditation training on mental health and well-being in the ageing population. The Age-Well RCT primary objective was to evaluate the effect of an 18-month meditation-based intervention in cognitively unimpaired older adults as compared with an 18-month non-native language (i.e., English) training program and a passive control group (no intervention).^17,18^ Individuals were recruited from the general population from November 2016 to May 2018 in Caen, France. Full exclusion/inclusion criteria presented in the protocol paper^17^ and the primary outcome paper^18^ are available in eTable 1. In brief, they were older than 65 years, native French speakers, retired for at least 1 year, had at least 7 years of education, and performed within the normal range on standardized cognitive tests. Owing to the characteristics of the trial, they must have no present or past regular or intensive practice of meditation or comparable practices, nor speaking fluent English. The main exclusion criteria consisted of a history of major neurologic or psychiatric disorders (e.g., substance abuse), the presence of a chronic illness or an acute unstable condition (e.g., cardiovascular or metabolic diseases), and current or recent treatments that might affect cognitive function. All participants underwent multimodal neuroimaging evaluations (e.g., structural MRI, dual-phase ^18^F-florbetapir-PET, and ^18^F-fluorodeoxyglucose (FDG)–PET scans), blood sampling, along with detailed clinical, cognitive, behavioral and sleep assessments, prior and after the interventions.

In this study, only baseline behavioral (lifestyle) and neuroimaging data, acquired prior randomization, were used.^17^

### Standard Protocol Approvals, Registrations, and Patient Consents

The Age-Well trial, sponsored by the Institut National de la Santé et de la Recherche Médicale (INSERM), was approved by the ethics committee (CPP Nord-Ouest III, Caen; Clinicaltrials.gov Identifier: NCT02977819; trial registration number: EudraCT: 2016-002441-36; IDRCB: 2016-A01767-44; registration date: November 25, 2016), and all participants gave their written informed consent to participate in the study.

### Lifetime of Experiences Questionnaire

The Lifetime of Experiences Questionnaire (LEQ),^19^ translated and adapted to the French culture in the context of the Medit-Ageing project,^20^ was used to assess participants' engagement in various lifestyle activities across 3 life periods: young adulthood (LEQ-young, 13–30 years), midlife (LEQ-midlife, 30–65 years), and late-life (LEQ-late, 65 years to present date). The entire questionnaire was self-completed by participants in 1 sitting, within the same session (during a behavioral examination session). While questionnaires were completed autonomously, a clinical neuropsychologist was available in case of questions. LEQ were checked by a neuropsychologist/psychometrist; when missing values were identified, the information was collected through phone calls.

For each life period, the LEQ score is subdivided into a specific and a non-specific score. More specifically, the LEQ specific scores represent educational experience (primary, secondary, and postsecondary education) in young adulthood, occupation complexity, managerial experience and additional education during midlife, and engagement in social, leisure, and information-seeking behaviors (e.g., How do you usually acquire your information about world and national events?) and additional education in late life. Non-specific scores combine activities that are similar for all life periods. They include a standard set of 7 questions about the participation in various leisure activities such as visits to family or friends, music practice, participation in artistic activities (e.g., drawing, painting, theatre), physical activities (mild, moderate and high intensity), reading, second language practice, and travelling. Lifestyle engagement in young adulthood, midlife, and late life were calculated by summing specific and non-specific scores. To allow for direct between-period (LEQ-young/LEQ-midlife/LEQ-late) or between-activity types (specific/non-specific) comparisons, each score was standardized in z-scores. Higher scores reflect greater engagement in lifestyle activities.

### Neuroimaging Data

Participants were all scanned on the same MRI (Philips Achieva 3.0T scanner) and PET (Discovery RX VCT 64 PET-CT scanner; General Electric Healthcare) cameras at the Cyceron Center (Caen, France). High-resolution T1-weighted anatomical volumes using a three-dimensional fast-field echo sequence (Sagittal 3D-FFE, repetition time [TR]/echo time [TE] = 7.1/3.3 ms, field of view [FOV] = 256 × 256 mm^2^, 180 slices, 1 × 1 × 1 mm^3^) and a three-dimensional fluid-attenuated inversion recovery image (FLAIR; sagittal; TR/TE 4.8/272 ms; inversion time = 1.650 ms; FOV = 250 × 250 mm^2^, 180 slices, voxel size: 0.98 × 0.98 × 1 mm^3^) were acquired in all participants. They were then segmented, normalized to the Montreal Neurologic Institute (MNI) template, and modulated to correct for nonlinear warping effects using the Statistical Parametric Mapping (SPM12) software.

FDG-PET was acquired after a 6-hour fasting period. After a resting period of 30 minutes in a quiet and dark environment, ∼180MBq of FDG were intravenously injected as a bolus. A 10-minute scan was acquired 50 minutes after injection. A dual-phase Florbetapir-PET scan was performed to measure brain perfusion (early acquisition) and amyloid burden (late acquisition). More specifically, a first 10-minute scan started at the time of the IV injection of ∼4MBq/kg of Florbetapir (early-Florbetapir-PET) and a second 10-minute scan was acquired 50 minutes after the injection (late-Florbetapir-PET). Each PET image was coregistered onto its corresponding MRI and normalized to the MNI template by applying the deformation parameters defined from the T1-weighted normalization procedure as described above. PET-Images were then quantitatively normalized using as a reference region either the brainstem for early Florbetapir-PET scans or the cerebellar gray matter for both late Florbetapir-PET scans and FDG-PET scans, resulting in standardized uptake value ratio (SUVR). Early Florbetapir-PET scans, corresponding to the brain's perfusion, were reconstructed from 1 to 5 minutes (included). Normalized MRI images were smoothed with a Gaussian kernel of 10 mm full width at half maximum and PET images were smoothed with a Gaussian kernel of 8 mm full width at half maximum to allow for voxel-wise analyses. FDG-PET was missing in 45 participants. Late Florbetapir-PET acquisition was missing in 1 participant, and early acquisition was missing in 1 additional participant because of a scanner issue.

To investigate more specifically regions that are sensitive to AD, we extracted the volume/SUVR in AD-signature regions. More specifically, the hippocampus, a region particularly sensitive to atrophy in AD, was segmented based on T1-weighted images using the Automatic Segmentation of Hippocampal Subfields (ASHS) software.^21^ Left and right volumes were averaged, and raw bilateral volumes were then normalized by the total intracranial volume (TIV) to account for interindividual variability in head size (volume x100/TIV). The TIV-normalized hippocampal volume was obtained from the ASHS segmentation. Concerning metabolic AD-signature regions, average glucose metabolism and cerebral perfusion were extracted from the temporoparietal cortex (metabolic AD-signature regions^22^) and neocortical amyloid burden was extracted from the entire gray matter, except the cerebellum, occipital and sensory motor cortices, hippocampi, amygdala, and basal nuclei for amyloid AD-signature region of interest.^23^

### Statistical Analyses

Correlations and descriptive analyses were performed on raw LEQ scores and subscores to provide greater insights on LEQ changes across lifespan.

**Figure 1:**
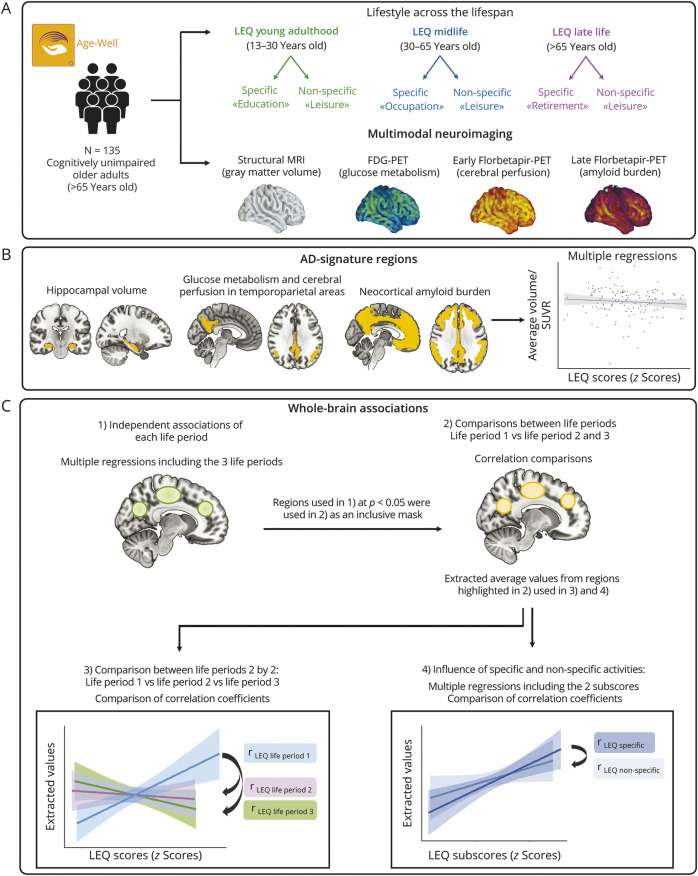
Design Overview (A) In this cross-sectional design, 135 cognitively unimpaired older adults from the Age-Well cohort completed the Lifetime of Experiences Questionnaire (LEQ), allowing to retrospectively evaluate their lifestyle during young adulthood, midlife, and late-life. A score was obtained for each life period and reflected both activities that were specific and non-specific to this period. Multimodal neuroimaging was also obtained including structural MRI (gray matter volume), FDG-PET (cerebral glucose metabolism), and Florbetapir-PET scans (early frames/cerebral perfusion and late frames/amyloid burden). (B) Multiple linear regressions were performed including LEQ scores for the 3 life periods as predictors of AD-signature regions (1 model per imaging modality; hippocampal volume, glucose metabolism, or cerebral perfusion in temporoparietal areas, neocortical amyloid burden), controlling for age and sex. (C) Whole brain analyses: (1) Voxel-wise multiple linear regressions were conducted to assess the association of LEQ score at each life period (in the same model) with neuroimaging (1 model per neuroimaging modality). Results were considered significant at *p* < 0.005 (uncorrected), k > 100 (Figure 1C1, green regions). (2) When an association was found between lifestyle at a given life period and imaging, we conducted additional analyses within an inclusive mask including the regions highlighted to further understand the specificity of this association and the type of activity that could drive this effect. More specifically, within the regions significant at *p* < 0.05 (used as an inclusive mask), we assessed voxel-wise whether the association with this life period was stronger than the association with the 2 other life periods. The average volume/SUVR of the regions found to be more related to this life period than to the 2 others (Figure 1C2, orange regions) were extracted to (3) conduct a 2-by-2 correlation coefficient comparisons between the 3 life periods and (4) assess the relative influence of specific and non-specific activities conducting comparisons of the correlation coefficient for specific vs non-specific subscores. LEQ = Lifetime of Experiences Questionnaire; SUVR = Standardized Uptake Value Ratio.

We conducted multiple linear regressions including, within the same model, the LEQ-scores for the 3 life periods as predictors of each neuroimaging modality, both in AD-sensitive regions (hippocampal volume, temporoparietal metabolism and perfusion, neocortical amyloid burden; Figure 1B) and voxel-wise (Figure 1C). The statistical approach is illustrated in Figure 1C.

For each life period-associated score showing an association with a given brain outcome, we performed voxel-wise comparisons between all period-specific scores and that specific brain outcome using an inclusive mask (p_uncorrected_ <0.05) (Figure 1C).

We then conducted confirmatory 2-by-2 correlation coefficient comparisons using R-studio's *cocor* package (Figure 1C).^24^ Of note, we applied Zou's (2007) method to compute a 95% CI for the difference between the 2 dependent correlations. Finally, to evaluate whether these associations were driven by 1 type of lifestyle activities, we performed multiple linear regressions and correlation comparisons between specific and non-specific subscores to assess their impact on previously identified brain outcomes (Figure 1C).

All analyses were conducted controlling for age, sex (self-reported), and the other LEQ periods (or the other subscore when specific and non-specific subscores were compared). The *cocor* method does not allow for the inclusion of covariates; consequently, analyses presented were conducted on the residuals from the linear association between brain outcomes and age, sex, and the other LEQ scores (either the other periods or type of activity) (supplementary results eTables 2–5). Analyses without residuals were also performed and results were largely similar (supplementary results eTables 6–9). Voxel-wise analyses were conducted using SPM12, and significance was set at p_uncorrected_ <0.005 and k > 100. The other analyses were conducted using R-studio, and results were considered significant at *p* < 0.05, without corrections for multiple comparisons.

### Data Availability

Data are available upon request following a formal data sharing agreement and approval by the Medit-Ageing consortium and executive committee. The data sharing request form can be downloaded online (silversantestudy.eu/2020/09/25/datasharing). The material can be mobilized, under the conditions and modalities defined in the Medit-Ageing Charter, by any research team belonging to an academic for performing a scientific research project relating to the scientific theme of mental health and well-being in older people. The material may also be mobilized by nonacademic third parties, under conditions, in particular financial, which will be established by separate agreement between INSERM and by the said third party. Data sharing policies described in the Medit-Ageing Charter are following our ethics approval and guidelines from our funding body.

## Results

Participants' characteristics are presented in Table 1. A total of 137 cognitively unimpaired older adults (older than 65 years) were included at baseline. Two participants were excluded for not meeting eligibility criteria. As a result, the analyses were performed on a sample of 135 participants older than 65 years who all underwent lifestyle assessments, along with multimodal neuroimaging at baseline (see Flow Chart, eFigure 1). Owing to missing data for some neuroimaging modalities (see Methods), analyses were conducted on 135 participants for gray matter volume (GMv; T1-weighted imaging), 92 participants for glucose metabolism (FDG-PET), 133 participants for cerebral perfusion (early-Florbetapir-PET), and 134 participants for amyloid burden (late-Florbetapir-PET).

**Table 1:** Participants' Characteristics

Characteristics	
Age, y	69.3 ± 3.79
Sex ratio, female/male	83/52
Education, y	13.16 ± 3.08
MMSE, score	29.04 ± 1.03
LEQ young adulthood, score	31.3 ± 9.3 (10–58.83)
LEQ specific young adulthood, score	19.87 ± 7.15 (0–31.5)
LEQ non-specific young adulthood, score	18.57 ± 3.05 (7–27)
LEQ midlife, score	38.44 ± 8.5 (21–59.2)
LEQ specific midlife, score	18.87 ± 7.15 (6–40.65)
LEQ non-specific midlife, score	18.57 ± 3.05 (13–27)
LEQ late-life, score	28.14 ± 4.5 (18–38.4)
LEQ specific late-life, score	10.4 ± 2.23 (6–18.4)
LEQ non-specific late-life, score	17.81 ± 3.28 (10–26)

Abbreviations: LEQ = Lifetime of Experiences Questionnaires; MMSE = Mini Mental State Examination.

Demographic and lifestyle variables of the study sample (n = 135). Values represent mean ± SD (range) or number of participants.

Descriptive analyses on raw LEQ scores indicated that all LEQ scores and subscores were correlated (*p* < 0.05) with the exception of the correlation between LEQ-young specific and late-life non-specific subscores (*p* = 0.09, *r* = 0.15; eFigure 2). Correlations between young adulthood, midlife, and late-life periods were weak to moderate (ranging from *r* = 0.32 to *r* = 0.48 for total scores, *r* = 0.17 to *r* = 0.41 for specific subscores, *r* = 0.36 to *r* = 0.52 for non-specific subscores), suggesting some variability in LEQ scores across life periods (eFigures 3–5). Despite these correlations, analyses including the different LEQ subscores in the same model were possible because no strong evidence of multicollinearity was observed (variance inflation factor <2, reflecting a weak multicollinearity between variables).

### Association Between Lifestyle at Different Life Periods and Neuroimaging

No associations were found between AD-signature regions and LEQ scores at any life period (all *p* > 0.25, Table 2).

**Table 2:** Association Between LEQ Scores for Each Life Period and AD-Signature Regions

	LEQ-young			LEQ-midlife			LEQ-late		
	b	*t*	*p* Value	b	*t*	*p* Value	b	*t*	*p* Value
Hippocampal volume	0.004	0.14	0.89	−0.01	−0.50	0.62	−0.007	−0.26	0.80
Temporoparietal glucose metabolism	0.015	0.40	0.69	0.016	0.77	0.44	−0.018	−0.38	0.70
Temporoparietal cerebral perfusion	<0.001	0.002	0.99	<0.001	−0.81	0.42	<0.001	0.38	0.71
Neocortical amyloid burden	0.009	0.55	0.58	−0.02	−1.16	0.25	−0.01	−0.75	0.45

Abbreviation: LEQ = Lifetime of Experiences Questionnaire.

Statistical values were obtained from multiple linear regressions including LEQ z-scores for the 3 life periods (in the same models) as predictors of neuroimaging, controlling for age and sex.

Voxel-wise multiple regressions revealed no association between the LEQ-young score and neuroimaging data.

**Figure 2:**
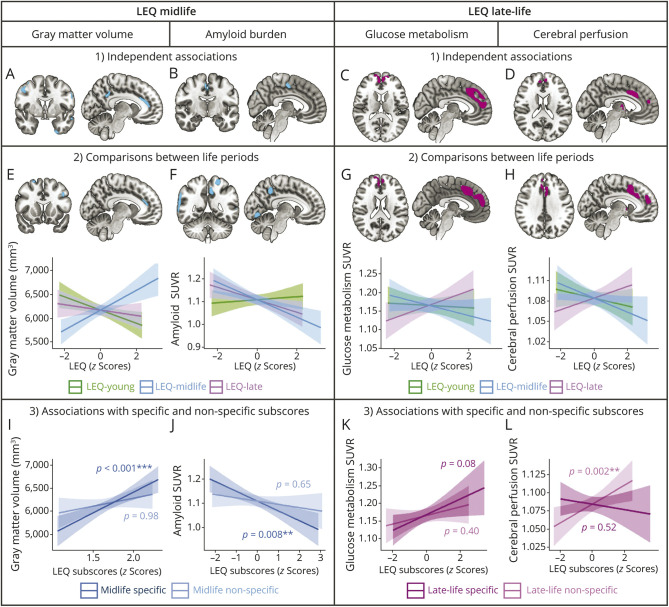
Associations Between Midlife and Late-Life LEQ Scores and Brain Integrity Left panel: (1) Voxel-wise associations between LEQ-midlife and gray matter volume (A) and amyloid burden (B), controlling for age, sex, and LEQ-young and LEQ-late scores. (2) Regions showing greater association with LEQ-midlife compared with the other 2 periods for gray matter volume (E) and amyloid burden (F) and associated graphic representations. (3) Associations between LEQ-midlife specific (dark blue) or non-specific (light blue) subscores with gray matter volume (I) and amyloid burden (J) in regions previously highlighted (panels E and F). Statistical values were obtained from multiple regression models including specific and non-specific subscores to predict the average volume/SUVR within the regions previously evidenced, controlling for age and sex. Right panel: (1) voxel-wise associations between LEQ-late and cerebral perfusion (G) and glucose metabolism (D), controlling for age, sex, and LEQ-young and LEQ-midlife scores. (2) Regions showing greater association with LEQ-late compared with the other 2 periods for cerebral perfusion (G) and glucose metabolism (H) and associated graphic representations. (3) Associations between LEQ-late-life specific (light pink) or non-specific (dark pink) subscores with glucose metabolism (K) and cerebral perfusion (L) in regions previously highlighted (panels G and H). Statistical values were obtained from multiple regression models including specific and non-specific subscores to predict the average volume/SUVR within the regions previously evidenced, controlling for age and sex. Voxel-wise results were considered significant at *p* < 0.005 uncorrected, k > 100. Linear regression lines and standard error (SE) bands corresponding to the 95% CI are represented. *p* < 0.05*, *p* < 0.01**, *p* < 0.001***. LEQ = Lifetime of Experiences Questionnaire; SUVR = Standardized Uptake Value Ratio.

Higher LEQ-midlife score was associated with higher GMv in the left anterior and middle cingulate cortices, middle and inferior temporal gyrus, middle and inferior frontal gyrus, the left insula and Heschl's gyrus, and in the right superior frontal gyrus (Figure 2A). Higher LEQ-midlife was also associated with lower amyloid burden in the right middle cingulate cortex, middle temporal pole, and cuneus (Figure 2B). No association was found between LEQ-midlife and glucose metabolism or cerebral perfusion.

Higher LEQ-late score was associated with higher cerebral perfusion in the left anterior and middle cingulate cortices and the superior medial frontal gyri (Figure 2C) and with higher glucose metabolism in the anterior and middle cingulate cortices and the superior medial frontal gyri and left thalamus (Figure 2D). The LEQ-late score was not associated with GMv or amyloid burden.

### Association Between LEQ-Midlife and GMv

#### Comparison of the Correlation With LEQ-Midlife vs the 2 Other Periods

To further assess the specificity of our results, we tested whether the association between LEQ-midlife score and GMv within the regions identified in voxel-wise analyses (Figure 2A; inclusive mask at p_uncorrected_ <0.05) was stronger than the association with the 2 other life periods. Voxel-wise comparisons showed a stronger association between the LEQ-midlife score and GMv compared with the other periods in the left anterior cingulate, the anterior, middle and inferior temporal gyrus, inferior and right superior frontal gyrus, the left insula, and the Heschl's gyrus (Figure 2E). Correlation coefficient comparisons between the average GMv of these regions and LEQ scores further indicated that GMv was more positively correlated with LEQ-midlife than with LEQ-late (*p* < 0.001, 95% CI [0.4940–0.8025]) and LEQ-young scores (*p* < 0.001, 95% CI [0.6561–0.9467], eTable 2). GMv was also more positively correlated with LEQ-late than with LEQ-young (*p* = 0.04, 95% CI [–0.0190 to 0.3279], eTable 2), but this was not significant without considering the residuals (*p* = 0.11, eTable 6).

#### Comparison of the Correlation With LEQ-Midlife Specific vs Non-specific Subscores

Multiple linear regression between the average GMv in the regions previously identified and the LEQ-midlife specific (i.e., *occupation and managerial experience*) and non-specific (i.e., *general leisure activities*) subscores indicated that higher LEQ-midlife specific subscore was associated with higher GMv (b = 254, *p* < 0.001), while the non-specific subscore was not (b = 1.68, *p* = 0.98; Figure 2I). Correlation coefficient comparisons further indicated that LEQ-midlife specific subscore was more strongly correlated with GMv than the non-specific one (*p* < 0.001, 95% CI [0.1292–0.5135], eTable 3).

### Association Between LEQ-Midlife and Amyloid Burden

#### Comparison of the Correlation With LEQ-Midlife vs the 2 Other Periods

The association between higher LEQ-midlife score and lower amyloid burden previously highlighted (Figure 2B; inclusive mask at p_uncorrected_ <0.05) was stronger than the association with the 2 other periods in the left precuneus and superior parietal gyrus, and the right middle and superior temporal gyrus (Figure 2F). Correlation coefficient comparisons between the average amyloid burden within these regions and LEQ scores further revealed that amyloid burden was more strongly associated with LEQ-midlife than LEQ-young (*p* < 0.001, 95% CI [–0.5908 to −0.2598]), but did not differ from the correlation with LEQ-late (*p* = 0.17, 95% CI [–0.2455 to 0.0863]), which was also higher than the correlation with LEQ-young (*p* < 0.001, 95% CI [–0.5374 to −0.1530], eTable 2).

#### Comparison of the Correlation With LEQ-Midlife Specific vs Non-specific Subscores

Lower amyloid burden (in the regions previously highlighted) was associated with higher LEQ-midlife specific subscore (i.e., *occupation and managerial experience*; b = −0.03, *p* = 0.008), but not with the non-specific one (i.e., *general leisure activities*; b = −0.005, *p* = 0.65; Figure 2J). Correlation coefficient comparisons further indicated that the LEQ-midlife specific subscore was more strongly associated with amyloid burden than the non-specific one (*p* < 0.05, 95% CI [–0.3669 to 0.0283], eTable 3).

### Association Between LEQ-Late-Life and Glucose Metabolism

#### Comparison of the Correlation With LEQ-Late vs the 2 Other Periods

The positive correlation between LEQ-late and glucose metabolism previously evidenced (Figure 2C; inclusive mask at p_uncorrected_ <0.05) was stronger than the correlation with the 2 other periods in the anterior and middle cingulate, and superior medial frontal gyri (Figure 2G). Correlation coefficient comparisons further indicated that the average glucose metabolism within these regions was more strongly associated with LEQ-late than it was with LEQ-young (*p* < 0.05, 95% CI [0.0704–0.5257]) or LEQ-midlife (*p* < 0.001, 95% CI [0.3180–0.7115]) (eTable 4). The average glucose metabolism within these regions was also more strongly associated with LEQ-young than it was with LEQ-midlife (*p* < 0.05, 95% CI [0.0202–0.4179], eTable 4A), but this was not significant without considering the residuals (*p* = 0.13, eTable 8).

#### Comparison of the Correlation With LEQ-Late Specific vs Non-Specific Subscores

Multiple linear regression showed no association between glucose metabolism (in the regions previously highlighted) and LEQ-late specific (b = 0.08, *p* = 0.08; Figure 2K) or non-specific (b = 0.009, *p* = 0.39) subscores. Correlation coefficient comparisons further revealed that the association with glucose metabolism did not differ between LEQ-late specific (i.e., *engagement in social, leisure, and information-seeking behaviors*) and non-specific (i.e., *general leisure activities*) subscores (*p* = 0.16, 95% CI [–0.1141 to 0.3322], eTable 5).

### Association Between LEQ-Late Life and Cerebral Perfusion

#### Comparison of the Correlation With LEQ-Late vs the 2 Other Periods

Voxel-wise comparisons showed that the positive correlation between LEQ-late and cerebral perfusion previously evidenced (Figure 2D; inclusive mask at p_uncorrected_ <0.05) was stronger than the correlation with the 2 other periods in the left anterior and middle cingulate cortex, superior medial frontal gyri, and thalamus (Figure 2H). Correlation coefficient comparisons further indicated that higher LEQ-late was more positively associated with higher perfusion in these regions than LEQ-young (*p* < 0.05, 95% CI [0.1748–0.5536]) or LEQ midlife (*p* < 0.001, 95% CI [0.3654–0.6883], eTable 4). Perfusion in these regions was also more positively associated with the LEQ-young than LEQ-midlife (*p* < 0.05, 95% CI [–0.0046 to 0.3296], eTable 4), but this result was not significant without considering the residuals (*p* = 0.19, eTable 8).

#### Comparison of the Correlation With LEQ-Late Specific vs Non-specific Subscores

Multiple linear regressions revealed that higher LEQ-late non-specific subscore was associated with higher cerebral perfusion in the regions previously identified (b = 0.016, *p* = 0.002), while LEQ-late specific subscore was not (b = −0.003, *p* = 0.52; Figure 2L). Correlation coefficient comparisons further indicated that LEQ-late non-specific subscore (i.e., *general leisure activities*) was more strongly associated with cerebral perfusion than the LEQ-late specific one (i.e., *engagement in social, leisure, and information-seeking behaviors, p* < 0.05, 95% CI [0.1063–0.4901], eTable 5).

## Discussion

Our study shows that midlife lifestyle engagement is more strongly associated with greater structural integrity, notably in the anterior cingulate cortex, and with lower amyloid burden in the precuneus than lifestyle in young and late-adulthood periods. Consistently, previous research found that midlife engagement in physical and cognitive activities had a protective role on brain structure and that a healthy midlife lifestyle was associated with lower amyloid burden and white matter lesions in healthy older adults.^14,25-27^ More specifically, our findings suggest that these associations are driven by occupation and managerial experience in midlife. Although our results were found in different brain regions, this aligns with a study showing that midlife managerial experience is related to a slower rate of hippocampal atrophy in healthy older adults.^28^ Some studies have already shown the positive influence of past cognitive activity (young adulthood and midlife) on amyloid burden in older adults.^15^ Our research suggests that this association could be driven by midlife engagement and especially occupation complexity. While longitudinal studies are mandatory to better interpret these findings, our results suggest that midlife lifestyle engagement, especially occupation complexity, may promote BR or BM,^1,2^ allowing older adults to resist against atrophy and amyloid accumulation.

Compared with the other life periods, late-life lifestyle engagement was more strongly associated with brain perfusion and glucose metabolism in medial frontal regions, suggesting that current lifestyle relates more closely to brain function. These results align with prior studies showing the advantages of having a healthy and rich lifestyle in late-life (e.g., practicing physical activity and/or mental activities) on older adults' brain health.^29,30^ Notably, cognitive engagement in late-life has previously been associated with higher glucose metabolism in the frontal cortex.^31^ Such higher perfusion/metabolism in medial frontal regions may reflect more efficient or active brain networks and indicate increased CR, promoted by current engagement in complex mental activities.^2^ One possibility is that such stimulating activities could act as “mental exercise”, which could help maintain brain functioning (“use it or lose it” hypothesis^32^). An alternative interpretation would be that greater brain functioning in late life could facilitate the engagement in lifestyle activities, but further studies, with more appropriate designs, are needed to conclude on causality. The association with cerebral perfusion was stronger for late-life non-specific than specific activities. This suggests that a combination of social, artistic, cognitive, and physical engagement in late-life could be linked to greater brain function. Of note, the late-life specific and non-specific activities are very close, making the distinction between them more complex than for the other periods. Future studies should then adopt more fine-grained approaches to better determine the specific type of activity that is more likely to promote brain function in late-life.

While young adulthood lifestyle, and notably education, is a widely acknowledged reserve proxy^33,34^ that has been associated with greater structural and functional brain integrity along with lower pathologic burden,^35-37^ our study found no association between young adulthood lifestyle and brain integrity. However, inconsistencies exist in previous literature,^38,39^ with some studies showing no relationship between education and the rate of change in atrophy-prone cortical regions.^40^ Although there is no known reason for the lack of effect of education, this could be partly because of sample characteristics (e.g., in our case highly educated and motivated unimpaired older adults). For instance, we could speculate that other factors, including lifestyle engagement during other life periods, may counterbalance education-related differences in brain integrity. In addition, because the LEQ is based on the recollection of past events, measurements could be particularly imprecise for the most distant periods. While educational attainment might not be massively affected, we cannot rule out the possibility that a recollection bias for the young adulthood period made this measure imprecise.

Overall, lifestyle at both midlife and late-life was associated with brain integrity in anterior cingulate and medial frontal areas. These regions, particularly affected in aging,^41,42^ have been associated with resilience mechanisms, including cognitive resilience^43^ and reserve proxies.^44-46^ Our results align with the hypothesis that lifestyle could influence brain integrity by promoting resilience mechanisms. While no associations were evidenced with AD-signature regions, the association found with amyloid burden in the precuneus, which could be among the earliest regions showing amyloid accumulation,^47,48^ suggests that lifestyle might also influence earliest AD-related changes in healthy population.

One of the main strengths of this study is the lifestyle assessment across adulthood (past/current lifestyle) in a well-characterized sample of older adults, enabling to better disentangle the relative influence of lifestyle at each life period, by considering lifestyle in the other life periods. In addition, the use of multimodal neuroimaging enables a more integrated view of the association between lifestyle and brain health and their underlying resilience mechanisms.

This study has also limitations. The LEQ is based on a subjective and retrospective report of the participants. This retrospective aspect can lead to uncertainties in the answers, especially for the most distant periods. Nevertheless, the LEQ has been validated previously using test-retest,^19,49,50^ and the inclusion of only cognitively unimpaired older adults in our study should limit recollection bias. The LEQ questionnaire does not assess occupational activities in young adulthood (before 30 years) and late-life (after 65 years). Therefore, the potential benefits of occupation at these periods could not be captured. Future studies are needed to overcome this limitation and better estimate the potential benefits of occupational activities across the lifespan. Moreover, the cross-sectional design prevents us from drawing conclusions about causality, highlighting the need for larger longitudinal studies to (1) confirm the findings with data that are not subject to recollection bias and (2) evaluate the directionality of the relationship between lifestyle and brain health. Finally, our participants are highly educated and motivated, with a relatively healthy lifestyle. Future studies are mandatory to address the generalizability of the results to more diverse populations and to deepen our knowledge on the influence of lifestyle engagement on other key aspects of healthy aging (e.g., cognition, well-being, including satisfaction with life).

Overall, our study suggests that lifestyle at different life periods might have complementary benefits on distinct markers of brain health in late adulthood. A greater engagement in complex mental activities in midlife, and especially those related to occupation, might help to promote BR (higher gray matter volume) and resistance to brain pathology (lower amyloid burden) in late-life, while current (i.e., late-life) lifestyle would be more directly related to brain function. This study provides a better understanding of the factors influencing brain aging and may guide recommendations in dementia prevention by identifying the proper life period(s) to target.

## Appendix Coinvestigators

**Table:**

Coinvestigators are listed at Neurology.org/N.

## Glossary

AD: Alzheimer disease
ASHS: Automatic Segmentation of Hippocampal Subfields
BM: brain maintenance
BR: brain reserve
CR: cognitive reserve
FOV: field of view
GMv: gray matter volume
LEQ: Lifetime of Experiences Questionnaire
RCT: randomized controlled trial
SPM12: Statistical Parametric Mapping
SUVR: standardized uptake value ratio
TIV: total intracranial volume
